# Spatial transcriptomics in development and disease

**DOI:** 10.1186/s43556-023-00144-0

**Published:** 2023-10-09

**Authors:** Ran Zhou, Gaoxia Yang, Yan Zhang, Yuan Wang

**Affiliations:** 1 Department of Neurosurgery, State Key Laboratory of Biotherapy and Cancer Center, West China Hospital, Sichuan University, Chengdu, 610041 China; 2 National Clinical Research Center for Geriatrics, West China Hospital, Sichuan University, Chengdu, 610041 Sichuan China

## Abstract

The proper functioning of diverse biological systems depends on the spatial organization of their cells, a critical factor for biological processes like shaping intricate tissue functions and precisely determining cell fate. Nonetheless, conventional bulk or single-cell RNA sequencing methods were incapable of simultaneously capturing both gene expression profiles and the spatial locations of cells. Hence, a multitude of spatially resolved technologies have emerged, offering a novel dimension for investigating regional gene expression, spatial domains, and interactions between cells. Spatial transcriptomics (ST) is a method that maps gene expression in tissue while preserving spatial information. It can reveal cellular heterogeneity, spatial organization and functional interactions in complex biological systems. ST can also complement and integrate with other omics methods to provide a more comprehensive and holistic view of biological systems at multiple levels of resolution. Since the advent of ST, new methods offering higher throughput and resolution have become available, holding significant potential to expedite fresh insights into comprehending biological complexity. Consequently, a rapid increase in associated research has occurred, using these technologies to unravel the spatial complexity during developmental processes or disease conditions. In this review, we summarize the recent advancement of ST in historical, technical, and application contexts. We compare different types of ST methods based on their principles and workflows, and present the bioinformatics tools for analyzing and integrating ST data with other modalities. We also highlight the applications of ST in various domains of biomedical research, especially development and diseases. Finally, we discuss the current limitations and challenges in the field, and propose the future directions of ST.

## Introduction

Just as no man is an island, a cell’s behavior and function are not isolated, but rather influenced by neighboring cells organized in a three-dimensional space to form a tissue or organ [1]. Spatial organization is crucial for organogenesis during development, and its disruption/reorganization is implicated in the onset, progression, and treatment of diseases [2]. Thus, it is important to investigate cellular function in a spatial context, which could offer a more comprehensive and holistic view of complex biological systems, reveal novel mechanisms of organ development and diseases, and lead to new strategies for diagnosis and therapy.

Transcriptomics offers a high-throughput method to examine gene expression differences during development and disease. However, conventional methods for transcriptomic analysis, such as bulk RNA sequencing (RNA-seq) or single-cell RNA sequencing (scRNA-seq) have limitations in preserving the spatial information of the cells within a tissue [3]. Bulk RNA sequencing averages the gene expression across all cells in a tissue sample, losing the cellular heterogeneity and spatial context. Single-cell RNA sequencing dissociates the cells from the tissue, allowing for high-resolution analysis of individual cell types, but at the cost of losing the positional information of the cells. Furthermore, without spatial information, conventional methods for transcriptomic analysis do not enable easy integration of transcriptomic data with other types of data, such as histological images, proteomics or metabolomics. Collectively, this underscores the necessity for more extensive in situ transcriptional analysis to gain insights into the functioning of intricate biological systems.

Lately, numerous spatial gene profiling technologies have emerged with the goal of uncovering molecular alterations in two-dimentional (2D) and three-dimentional (3D) tissue samples at specific locations, and even achieving resolutions down to single cells or subcellular levels. The modern age technologies are originated from historical tools that enable simultaneous quantification of gene expression while retaining spatial information. The initial concept of in situ hybridization (ISH) dates back to 1969, when it was first employed for detecting DNA-RNA hybrids [4]. Subsequently, various iterations of this method have been extensively used to visualize gene expression at the single-cell and subcellular levels across space. Evolving further, in situ sequencing (ISS) generates a spatial transcriptome through sequencing by ligation (SBL), gene barcoding, or sequencing by oligonucleotide ligation and detection (SOLiD). Additionally, spatial information can be obtained by targeting and isolating specific regions of interest (ROIs) using methods like physical or optical marking. Following this, the isolated ROIs can be subjected to analysis through approaches like microarray, RNA-seq, or they can be dissociated for single-cell RNA-seq analysis.

While both in situ and ROI-based methods offer spatially resolved gene profiling, they fail to meet the requirement for rapid and high-throughput spatial profiling. More recently, an advanced technique called Spatial Transcriptomics (ST) has emerged, capable of mapping gene expression in tissues by integrating molecular profiling with spatial information [5]. It overcomes the limitations of conventional transcriptomic methods by assigning cell types (identified by their mRNA signatures) to their precise locations in histological sections, and can even determine subcellular localization of mRNA molecules. This enables us to uncover cellular heterogeneity and intercellular communications within a complex tissue/organ, providing an atlas for organ development and disease progression. Its power and potential have been recognized by *Nature Methods*, which declared ST as the method of the year 2020 [6].

Currently, this field is undergoing rapid expansion, driven by several factors including the decreasing costs associated with sequencing, collaborative initiatives undertaken by international consortiums, and notable progress in computing and imaging technologies. Consequently, gene expression landscapes with spatial resolution across a range of tissues, species, developmental stages, and disease conditions have been constructed. In this review, we aim to put everything in context and provide a comprehensive overview of the historical, technical, and application aspects of ST. We categorize and compare different types of ST methods based on their underlying principles and workflows, and we present the bioinformatics tools for analyzing and integrating ST data with other modalities. We also showcase the applications of ST in various domains of biomedical research, such as developmental biology, neuroscience, immunology, and oncology. Finally, we discuss the current limitations and challenges in the field, and we envision the future directions of spatial transcriptomics.

### Putting ST in a historical context: the development of a tapestry of methods

**Fig. 1:**
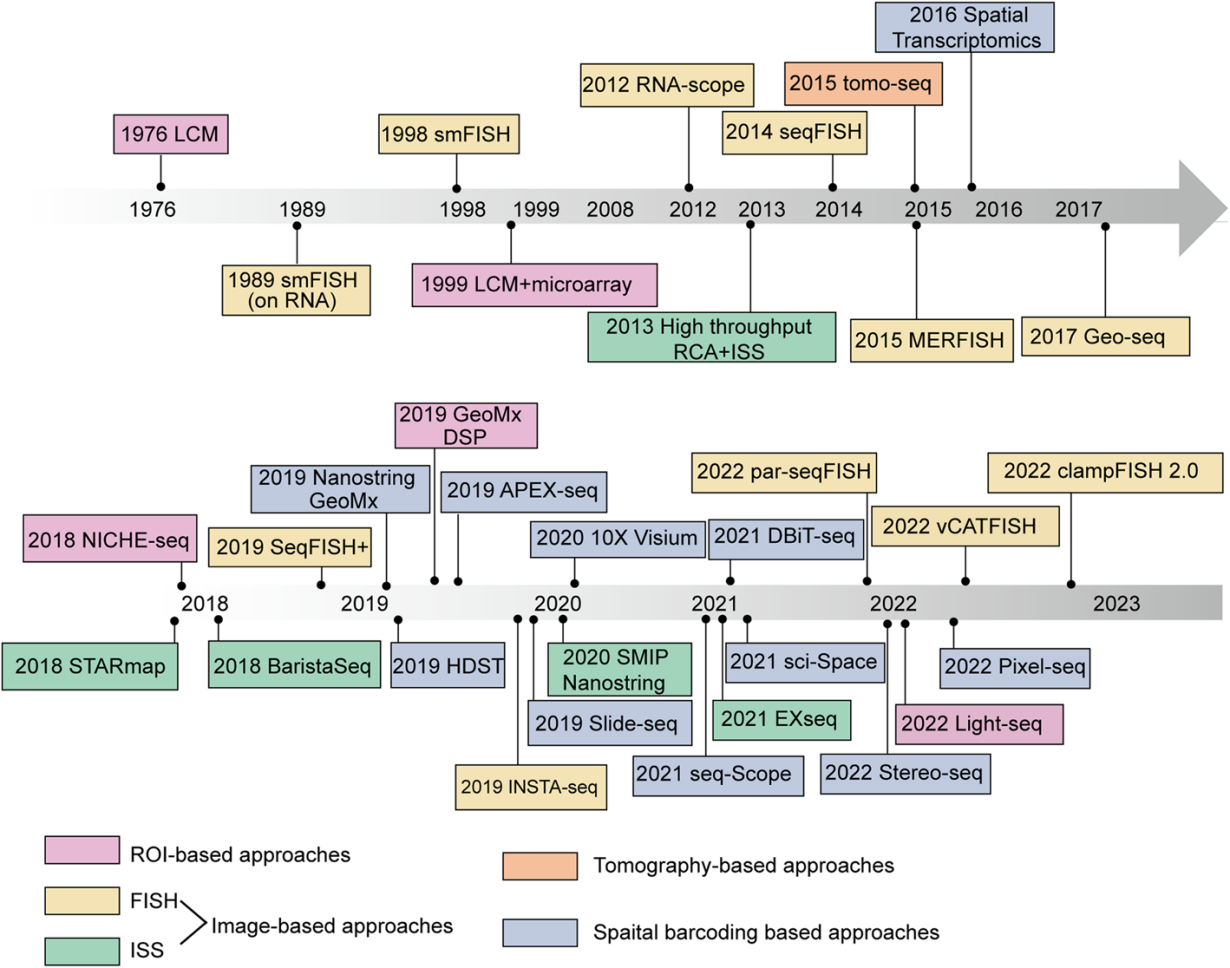
Timeline of the major published methods mentioned in this review. Every approach is categorized according to its foundational methodology, primarily segmented into the subsequent classifications: ROI-based methods (purple), image-based methods including FISH (yellow) and ISS (green), tomography-based methods (orange), and spatial barcoding-based methods (blue)

**Fig. 2:**
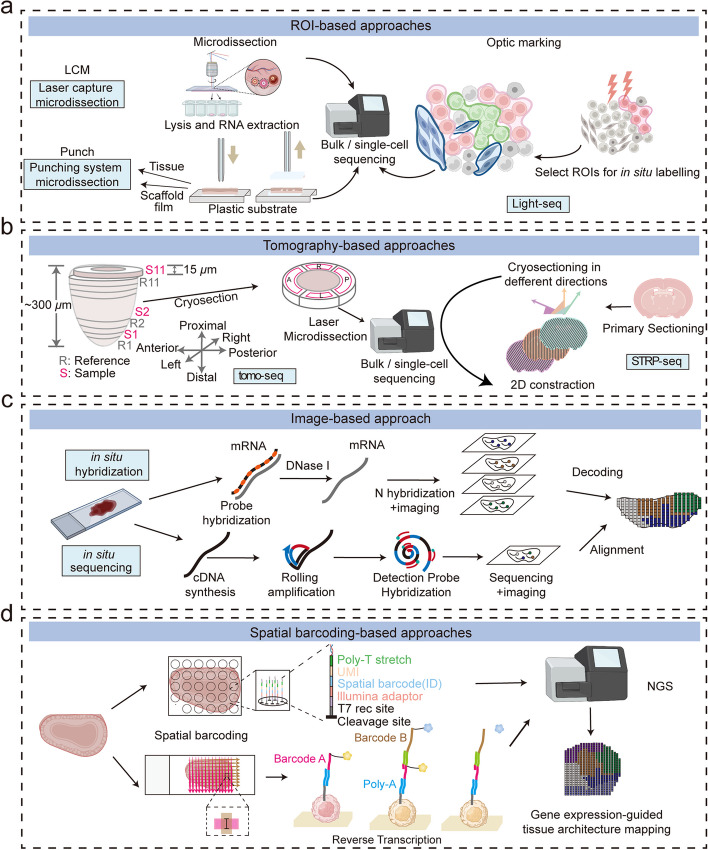
Overview of technologies for spatial transcriptomics. **a** Physical dissection or optical marking-based approaches involve the selection or marking of regions of interest. Following ROI marking, samples can be individually collected for RNA-seq or dissociated into single cells for scRNA-seq. **b** Image-based technologies achieve spatially resolved gene expression through decoding fluorescence signals. In situ sequencing and single molecule fluorescence in situ hybridization detect molecular abundance by directly reading transcript sequences within the tissue or target RNA barcodes, respectively. **c** Tomography-based methods, such as RNA tomo-seq, utilize a frozen section technique to linearly amplify cDNA from single tissue samples. Three identical biological samples are systematically frozen and sliced in three different directions, allowing for the completion of 2D transcriptional reconstruction through overlapping data. **d** Spatial barcoding-based methods generate spatial transcriptomes using reverse transcription primers with unique positional barcodes

Although ST has only become the buzz word in recent years, the idea of visualizing and quantify RNA expression in space is not entirely new. It can be traced back to the 1970s, when radioactive in situ hybridization (ISH) and laser capture microdissection (LCM) were first developed [5] (Fig. 1). These two methods, along with their various derivatives, have been used for decades to study spatial gene expression. They represent two schools of thoughts to obtain spatial information: to detect RNA molecules/sequences in their original location and match them with histological images, or to isolate ROIs within a defined spatial location to perform subsequent RNA analysis. These led to two categories of current ST methods: ROI-based approaches and in situ image-based approaches [5] (Fig. 2, Table 1). The advent of next generation sequencing (NGS) further expands the 2D ROI-based approaches to 3D tomography-based approaches, allowing for high-throughput whole tissue/organ profiling (Fig. 2, Table 1). In contrast, spatial barcoding approaches and spatial multi-omics are relatively new additions to the ST toolbox, which integrates localized spatial barcoding of RNA molecules in situ and NGS sequencing [5] (Fig. 2, Table 1).

**Table 1:** A detailed comparison of present methods for spatial transcriptomics

Method	Separation technique	Gene detection method	Throughput	Spatial resolution	Image context
punch	ROI/microdissection	NGS	Transcriptome-wide	N/A	ROI
SMD-seq	ROI/microdissection	NGS	Transcriptome-wide	N/A	ROI
Pick-Seq	ROI/microdissection	NGS	Transcriptome-wide	N/A	ROI
Select-seq	ROI/microdissection	NGS	Transcriptome-wide	N/A	ROI
Light-Seq	ROI/optocal marking	NGS	Transcriptome-wide	N/A	ROI
TIVA	ROI/optocal marking	NGS	Transcriptome-wide	N/A	ROI
NICHE-seq	ROI/optocal marking	NGS	Transcriptome-wide	N/A	ROI
GeoMX DSP	ROI/optocal marking	NGS	Transcriptome-wide	N/A	ROI
ZipSeq	ROI/optocal marking	NGS	Transcriptome-wide	N/A	ROI
PIC	ROI/optocal marking	NGS	Transcriptome-wide	N/A	ROI
SPACECAT	ROI/optocal marking	NGS	Transcriptome-wide	N/A	ROI
OpTAG-seq	ROI/optocal marking	NGS	Transcriptome-wide	N/A	ROI
TATTOO-seq	ROI/optocal marking	NGS	Transcriptome-wide	N/A	ROI
GeoMX SPG	ROI/optocal marking	NGS	Transcriptome-wide	N/A	ROI
Geo-seq	Tomography	NGS	Transcriptome-wide	N/A	whole tissue / orgran
Cryo-Sliced	Tomography	NGS	Transcriptome-wide	N/A	whole tissue / orgran
Transcriptome Tomography	Tomography	NGS	Transcriptome-wide	N/A	whole tissue / orgran
Tomo-seq	Tomography	NGS	Transcriptome-wide	N/A	whole tissue / orgran
STRP-seq	Tomography	NGS	Transcriptome-wide	N/A	whole tissue / orgran
SRM seqFISH	image	FISH	Predefined gene probes	subcellular	whole tissue
seqFISH	image	FISH	Predefined gene probes	subcellular	whole tissue
MERFISH	image	FISH	Predefined gene probes	subcellular	whole tissue
ExM-MERFISH	image	FISH	Predefined gene probes	subcellular	whole tissue
seqFISH+	image	FISH	Predefined gene probes	subcellular	whole tissue
ISS	image	ISS	Predefined gene probes	subcellular	whole tissue
FISSEQ	image	ISS	Transcriptome-wide	subcellular	whole tissue
STARmap	image	ISS	Predefined gene probes	subcellular	whole tissue
INSTA-seq	image	ISS	Predefined gene probes	subcellular	whole tissue
HybISS	image	ISS	Predefined gene probes	subcellular	whole tissue
ExSeq	image	ISS	Transcriptome-wide	subcellular	whole tissue
BOLORAMIS	image	ISS	Predefined gene probes	subcellular	whole tissue
STARmap PLUS	image	ISS	Predefined gene probes	subcellular	whole tissue
HybRISS	image	ISS	Predefined gene probes	subcellular	whole tissue
TEMPOmap	image	ISS	Predefined gene probes	subcellular	whole tissue
Xenium	image	ISS	Predefined gene probes	subcellular	whole tissue
Seq-Scope	spatial barcoding	NGS	Transcriptome-wide	<1 μm	whole tissue
Stereo-seq	spatial barcoding	NGS	Transcriptome-wide	0.22 μm	whole tissue
scStereo-seq	spatial barcoding	NGS	Transcriptome-wide	0.22 μm	whole tissue
Slide-TCR-seq	spatial barcoding	NGS	Transcriptome-wide	10 μm	whole tissue
Slide-seq	spatial barcoding	NGS	Transcriptome-wide	10 μm	whole tissue
DBiT-seq	spatial barcoding	NGS	Transcriptome-wide	10 μm	whole tissue
Slide-seqV2	spatial barcoding	NGS	Transcriptome-wide	10 μm	whole tissue
Matrix-seq	spatial barcoding	NGS	Transcriptome-wide	10 μm	whole tissue
ST	spatial barcoding	NGS	Transcriptome-wide	100 μm	whole tissue
SM-Omics	spatial barcoding	NGS	Transcriptome-wide	100 μm	whole tissue
sci-Space	spatial barcoding	NGS	Transcriptome-wide	118.2 ~ 174.6 μm	whole tissue
HDST	spatial barcoding	NGS	Transcriptome-wide	2 μm	whole tissue
Spatial ATAC–RNA-seq	spatial barcoding	NGS	Transcriptome-wide	20 μm	whole tissue
spatial-ATAC-seq	spatial barcoding	NGS	Transcriptome-wide	25 μm	whole tissue
spatial-CITE-seq	spatial barcoding	NGS	Transcriptome-wide	25 μm	whole tissue
MISAR-seq	spatial barcoding	NGS	Transcriptome-wide	50 μm	whole tissue
xDbit	spatial barcoding	NGS	Transcriptome-wide	50 μm	whole tissue
CBSST-Seq	spatial barcoding	NGS	Transcriptome-wide	50 μm	whole tissue
XYZseq	spatial barcoding	NGS	Transcriptome-wide	500 μm	whole tissue
SPOTS	spatial barcoding	NGS	Transcriptome-wide	55 µm	whole tissue

### ROI-based approaches

ROI-based approaches involve isolating specific ROIs within a sample through physical microdissection or optical marking. Once the ROIs are collected, they can be subjected to bulk RNA-seq, or dissociated into single cells for scRNA-seq [7–9]. Physical microdissection methods can isolate ROIs within samples using different techniques, such as LCM and micro-dissection punching system. LCM is a histology-based technique that uses either ultraviolet (UV) cutting or infrared (IR) capture systems to collect the desired ROI from tissue sections. SRS microdissection and sequencing (SMD-seq) [10] takes a step further to combine LCM with stimulated Raman scattering (SRS) microscopy, which provides chemical contrast to reveal histological tissue architecture without staining to guide the dissection of ROIs. Another approach for isolating ROIs within tissues is the micro-dissection punching system, which uses a punching unit to automatically collect tissue samples, allowing for quick and efficient collection of ROIs.

Optical marking methods can label ROIs using photoactivatable cell tagging or photoactivatable oligonucleotides. One way is to use genetically engineered mice expressing photoactivatable fluorescent markers in specific ROIs or cell types, such as NICHE-seq [11] and TATTOO-seq [12]. In contrast, SPACECAT [13] and OpTAG-seq [14] enable optical tagging of live cells for scRNA-seq without exogenous genes. Another approach is using oligonucleotide-antibody conjugates to put the spatial barcodes onto cell surface. ZipSeq [15] attaches the photo-uncaging barcodes onto the cell surface by the antibody or lipid DNA conjugate, and the spatial barcodes are uncaged and captured by 10 × Genomics scRNA-seq pipeline. Alternatively, Merritt et al. provide an integrated commercial system (GeoMX DSP) that enables highly multiplex spatial profiling of protein or RNA in FFPE tissue by oligonucleotide-conjugated RNA probes [16].

Instead of labeling cells directly, TIVA-tag [17] is an engineered method that allows for the annealing of mRNA from ROIs in live tissue through photoactivation. The mRNA tagged with TIVA can then be purified for downstream analysis. Along this line, Photo-isolation chemistry (PIC) [18] and Light-Seq [19] combine in situ reverse transcription (RT) with caged RT primers that are uncaged after UV irradiation of ROIs to generate spatially indexed sequencing libraries to integrate spatial information with gene expression.

### Tomography-based approaches

Tomography, a technique widely applied in medical imaging, entails the reconstruction of three-dimensional structures using a sequence of two-dimensional images captured from varying angles. In the context of spatial genomics, innovative methods like Tomo-seq [20], Cryo-Sliced [21], and Transcriptome Tomography [22] employ tomography principles to divide the entire tissue, organ, or embryo into multiple thin slices along different axes. Subsequently, RNA-seq analysis is conducted on each of these sections to gain insights into gene expression patterns. Sequential image optimization along different sectioning axes and iterative proportional fitting analysis are performed to mathematically reconstruct 3D expression images. Geo-seq [23] further integrates Tomo-Seq with LCM to achieve 3D spatial transcriptome by sequencing ROIs from different geographical positions. More recently, STRP-seq [24] offers a new imaging-free framework by slicing adjacent sections into multi-angle sections and reconstruct complex spatial patterns with an associated algorithm. The tomography-based approach offers a convenient and imaging-free technique for exploring spatial genomics. Nonetheless, this method is often characterized by its comparatively lower resolution in comparison to imaging-based approaches. Additionally, it may not fully capture intricate patterns like discontinuous or checkboard-like structures.

### Image-based approaches

Despite the power of ROI-based ST methods, they may introduce biases or errors in the spatial information due to the choice or quality of the ROIs. Moreover, ROI-based methods may not capture the continuous or gradual changes in gene expression across space, but rather discretize them into discrete ROIs. In situ image-based approaches overcome these limitations by probing or sequencing RNA molecules in situ on tissue sections to better preserve the spatial information, including single-molecular FISH (smFISH) and ISS.

SmFISH is a technique that uses fluorescent probes to image specific RNA transcripts in tissue sections. However, the number of genes that can be simultaneously imaged by conventional smFISH is limited by the number of spectrally distinct dyes. To overcome this limitation, several methods have been developed that use sequential barcoding or binary encoding schemes to increase the multiplexing capacity of smFISH [25–27]. For example, sequential fluorescent in situ hybridization (seqFISH) and its improved version seqFISH + use the same FISH probes for hybridization, but each round is labeled with a different fluorescent dye. With eight rounds of hybridization and four dyes, they can cover the entire transcriptome of human or mouse cells (4^8^ = 65,536). Another method, multiplexed error-robust FISH (MERFISH) [28], uses a binary encoding scheme in which the valid encoding words must have at least four Hamming distance from each other. This allows single-bit errors to be detected and corrected by comparing the observed words with a predefined cookbook. However, as the number of hybridization rounds increases, dropout events and spectral overlap may occur more frequently, which may affect the accuracy of target identification. To address this issue, some methods have integrated smFISH with expansion microscopy (ExM), which physically expands the tissue sample and increases the RNA density limit. For instance, ExM-MERFISH [29] and seqFISH + combined with ExM can substantially increase the number of molecules that can be detected without imaging crowding.

ISS is a technique that uses padlock probes and rolling circle amplification (RCA) to amplify and sequence gene barcodes or short fragments of cDNA directly in tissue sections [30]. Several methods have been developed based on ISS to achieve spatially resolved gene profiling. For example, ISS and barcoded oligonucleotides ligated on RNA amplified for multiplexed and parallel in situ analyses (BOLORAMIS) [31] used sequencing by ligation to read short segments of RNA from clonally amplified rolling-circle products (RCPs). The improved versions of ISS increased the efficiency of target detection by using different probe designs and signal amplification strategies [32, 33]. The spatially resolved transcript amplicon readout mapping (STARmap) [34] as well as STARmap PLUS [35] integrated hydrogel-tissue chemistry, targeted signal amplification, and in situ sequencing by dynamic annealing and ligation (SEDAL). A later adaptation with tri-probes (splint, primer and padlock) to detect metabolically-labeled RNAs was called TEMPOmap (temporally resolved in situ sequencing and mapping) [36].

Instead of detecting pre-designed gene barcodes, fluorescent in situ sequencing (FISSEQ) [37] as well as ExSeq [38], used SOLiD method to sequence circularized and RCA-amplified cDNAs. The next-generation of FISSEQ, termed INSTA-seq [39], efficiently imaged transcript-specific barcodes in situ and then assembled longer RNA molecules by using NGS. Although ISS was commercialized by Cartana, ISS-based methods are often technically challenging to implement due to the complex postprocessing and costly equipment.

### Spatial barcoding-based approaches

Although ROI- and image-based provide spatially resolved gene profiling, it can’t satisfy the demand of rapid and high-throughput spatial profiling. Spatial barcoding approach is a high-throughput technique that uses arrays of barcoded oligonucleotides to capture and sequence RNA molecules from tissue sections while preserving their spatial coordinates.

Several methods have been developed based on spatial barcoding to achieve high-throughput and genome-wide spatially resolved gene profiling. In 2016, Ståhl et al. first introduced spatial barcoding-based ST by positioning histological sections on arrayed reverse transcription primers with unique positional barcodes to generate RNA-seq data with two-dimensional positional information [40]. Its commercial version, Visium, has 55 µm-diameter spots in a hexagonal array, with center-to-center distance at 100 µm. Similarly, Slide-seq [41] and Slide-seqV2 [42] used uniquely DNA-barcoded 10 μm microparticles to capture RNA from tissue sections, and High-Definition Spatial Transcriptomics (HDST) deposited barcoded poly(d)T oligonucleotides into 2 μm wells [43].

More recently, spatial barcoding-based approaches have achieved subcellular resolution. PIXEL-seq achieved a spot diameter of about 1.22 μm [44], while Stereo-seq, scStereo-seq, Seq-Scope further reduced the spot sizes to sub-micrometer resolution [45–47]. Other spatial barcoding ST methods such as DBiT-seq [48], Matrix-seq [49], MISAR-seq [50], xDbit [51] and CBSST-Seq [52] use microfluidic-based methods to generated the spatial barcodes, allowing for spatial sequencing at pixel-size resolution. Still, none of these methods could ascertain that only a single cell was labelled by each barcode. XYZeq [53] and sci-Space [54] partially overcome this limitation by first spatially barcoding thousands of cells on the slide, and then subjecting them to scRNA-seq, revealing spatially expressed genes across cell types.

Transcriptomic analysis only reveals one aspect of cellular function. Spatial multi-omics is a technique that combines spatial transcriptomics with other omics modalities to reveal the multiple layers of cell function. Several methods have been developed to achieve comprehensive spatially resolved molecular profiling. For example, some methods have integrated antibody panels with transcriptomics, such as DBiT-Seq [48], GeoMX SPG [55], SM-Omics [56], spatial-CITE-seq [57], SPOTS [58] and Xenium [59]. These methods can simultaneously measure protein and RNA expression in tissue sections. For chromatin accessibility, some methods have adapted spatial transcriptomics with assay for transposase-accessible chromatin using sequencing (ATAC-seq), such as MISAR-Seq [50] and Spatial ATAC–RNA-seq [60]. These methods can simultaneously measure RNA expression and chromatin accessibility in tissue sections. For T-cell receptor (TCR) sequencing, a method called Slide-TCR-seq has combined RNase H-dependent PCR-enabled TCR sequencing (rhTCR-seq) with Slide-seq to simultaneously profile whole transcriptomes and TCRs within intact tissues [61].

### Putting the data together in a spatial context: analytical approaches for ST

**Fig. 3:**
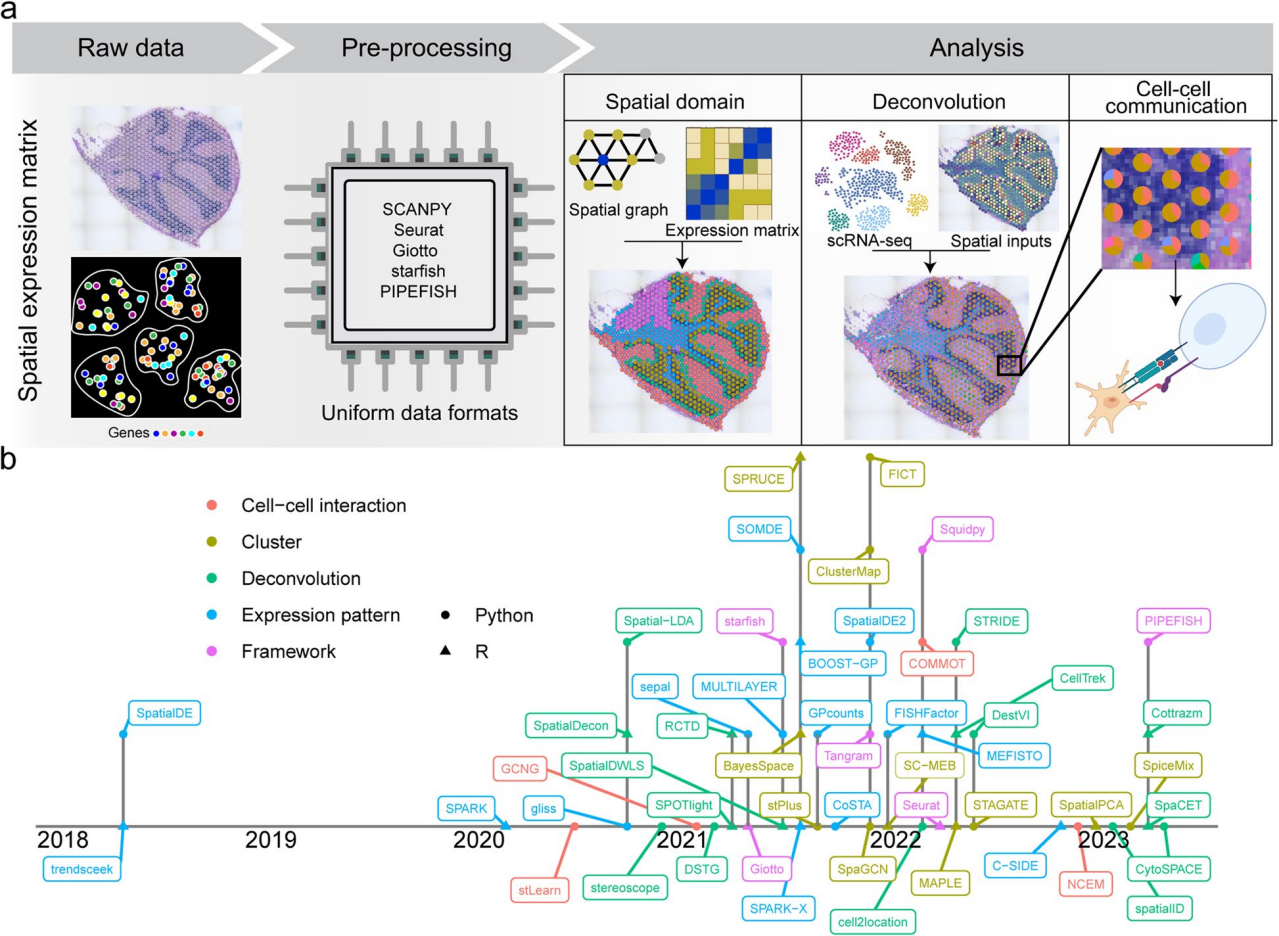
Overview of spatial transcriptomics tasks and analysis tools. **a** The analysis framework standardizes diverse spatial molecular datasets into a consistent data format, followed by detecting spatial domains, deconvoluting spots, and analyzing cell–cell communication. **b** Spatial analysis tool timeline showcasing task categories through color coding, with dot shapes representing the language used by each tool

High-throughput spatial transcriptomics data pose new computational challenges and opportunities that require novel methods and tools. Some of the key problems include data preprocessing, cell-type deconvolution, identification of spatially variable genes, and inference of cell–cell interactions (Fig. 3).

### Pre-processing spatial transcriptomic data

The pre-processing of the spatial transcriptomic data usually involves converting the raw signal into spatially resolved expression matrix and the steps required vary between technologies. For imaging-based spatial transcriptome data such as smFISH and ISS, several steps are involved in transforming imaging-based spatial transcriptome data into an expression matrix. Initially, the 5-dimensional images (including x, y, z, rounds, and channels) undergo pre-processing, including filtering to improve the signal-to-noise ratio and image registration. The transcripts are next recognized within every image using spot detection and signal decoding. This generates 3-dimensional data that contains the x and y coordinates and their respective intensity. Lastly, the cell boundaries are identified, and the readouts are assigned to the corresponding cells. As there are variations across different smFISH-based protocols, lots of analysis in published research rely on in-house code that has limited documentation. To address this issue, the starfish package [62] and an enhanced version called PIPEFISH [63] have been introduced to offer a detailed and broader analysis framework, and these help in the conversion of raw images into spatially resolved gene expression. In addition, the commercial platform named MERSCOPE and Xenium also provides a dedicated pre-processing pipeline.

For spatial barcoding-based methods such as Visium and other commercial platforms, they provide a graphical user interface (GUI) which allows for spatial barcode registration and spot pre-filtering and then user-friendly pre-processing pipeline is subsequently used to generate spatial gene matrix. Once the users obtain the expression matrix using method-specific pipelines, they can conduct downstream analysis using popular packages like Giotto [64], Seurat [65], and Squidpy [66]. These packages offer a unified format and an in-depth analysis platform including quality control (e.g., filtering poorly expressed gene or spots), comprehensive visualization, unbiased clustering as well as dimensionality reductions.

### Cell-type deconvolution

The lack of single-cell resolution in many existing spatially resolved methods makes it difficult to determine the spatial distributions of different cell types. Although a few methods have been achieved for deconvolution of bulk RNA-seq [67], there are some limitations for directly applying it to spatial transcriptomic data. Despite the pixel size of some methods can approach the size of mammalian cells, an individual spot of spatial transcriptomic data could overlap with multiple cells. Numerous tools have been developed to address this issue. For example, NMFreg [41], SpatialDWLS [68] and SPOTlight [69] decomposed spot transcriptomes into cell abundance by non-negative matrix factorization. Alternative tools based on probabilistic models such as stereoscope [70], SpatialDecon [71], RCTD [72], DestVI [73], STRIDE [74], SpaCET [75], Spatial-LDA [76], Cell2location [77] and Cottrazm [78] deconvolute the cellular proportion of the spots in cooperating with the gene signatures from scRNA-seq datasets. Some other tools like Spatial-ID [79], CellTrek [80], Tangram [81], DSTG [82] and CytoSPACE [83] utilize graph network or deep learning model to reconstruct the spatial cellular map by integrating single-cell and spatial transcriptomic data. Two recent studies evaluated the performance of various methods for cellular deconvolution of spatial transcriptomic data, providing users with the necessary information to select the method that best meets their needs [84, 85].

### Identification of spatial variable features

An initial step to link spatial features with biological significance is to identify genes that are enriched in specific spatial domains. Several methods have been developed to identify spatially variable genes, such as trendsceek [86], SpatialDE [87]/SpatialDE2 [88], SPARK [89]/SPARK-X [90], sepal [91], MULTILAYER [92], gliss [93], GPcounts [94], BOOST-GP [95], CoSTA [96], SOMDE [97], FISHFactor [98] and MEFISTO [99], these methods evaluate each gene individually and provide a *p*-value to indicate the spatial variability of a gene. Typically, these widely used tools involve unbiased spatial domain detection based on the intrinsic variance of gene expression. However, these approaches do not account for the neighboring similarity of cells or spots in spatial domains. As a result, the spatial expression patterns of the genes identified by these methods are not always guaranteed.

Several new methods have emerged for detecting spatial domains from spatial transcriptomic data. BayesSpace [100], SC-MEB [101] and SPRUCE [102] enhance the resolution of clustering analysis by bayesian mixture model-based methods. FICT [103], SpiceMix [104], stPlus [105], ClusterMap [106] and SpatialPCA [107] enhance the resolution of clustering analysis by incorporating spatial neighborhoods. SpaGCN [108], STAGATE [109] and MAPLE [110] implement a deep learning approach that integrate gene expression, spatial location as well as histology to identify spatial domains. stLearn [111] detects spatial domains using spatial location and morphological features to normalize gene expression data.

Due to lack of single-cell resolution among some widely used spatial protocols, the identification of spatially variable genes or domains will be biased by spatial cellular abundance. While deconvolution analysis can determine the cellular abundance in spatial transcriptomic data, current methods do not consider cellular proportions for searching spatially variable genes. In the context of mixtures of cell types, a recently developed tool called cell type-specific inference of DE (C-SIDE) employs a general parametric statistical model to estimate spatially variable genes with stratifying by cell type [112].

### Deciphering cell-to-cell communication

Ligand-receptor-mediated intercellular interactions play a critical role in organismal development and homeostasis. Typically, these cell-to-cell interaction tools infer the potential interaction based on the ligand-receptor pairs such as CellPhoneDB [113] as well as NicheNet [114]. However, existing models for inferring intercellular communication in tissues rely on molecular profiles of dissociated cells, without considering their spatial proximity. Soon afterward, GCNG [115] and node-centric expression models (NCEM) [116] utilize graph convolutional neural networks (GCNs) to modeling cellular neighborhood graph in cooperating with the pairwise expression of genes to predict cellular interaction across space. An alternative approach named COMMOT (COMMunication analysis by Optimal Transport) use the collective optimal to infer the cell-to-cell communications based on ligand-receptor pairs [117].

### Putting ST in the development and disease context

The rapid development of ST methods and analytic tools has enabled us to study the gene expression and cell function in 3D tissue/organs at an unprecedented resolution. We can now measure the spatial coordinates and molecular profiles of thousands of cells or even single cells within a tissue/organ, and reveal how they are organized and interact with each other. This allows us to better understand the complex biological processes that occur during development and disease.

### Embryonic development

**Fig. 4:**
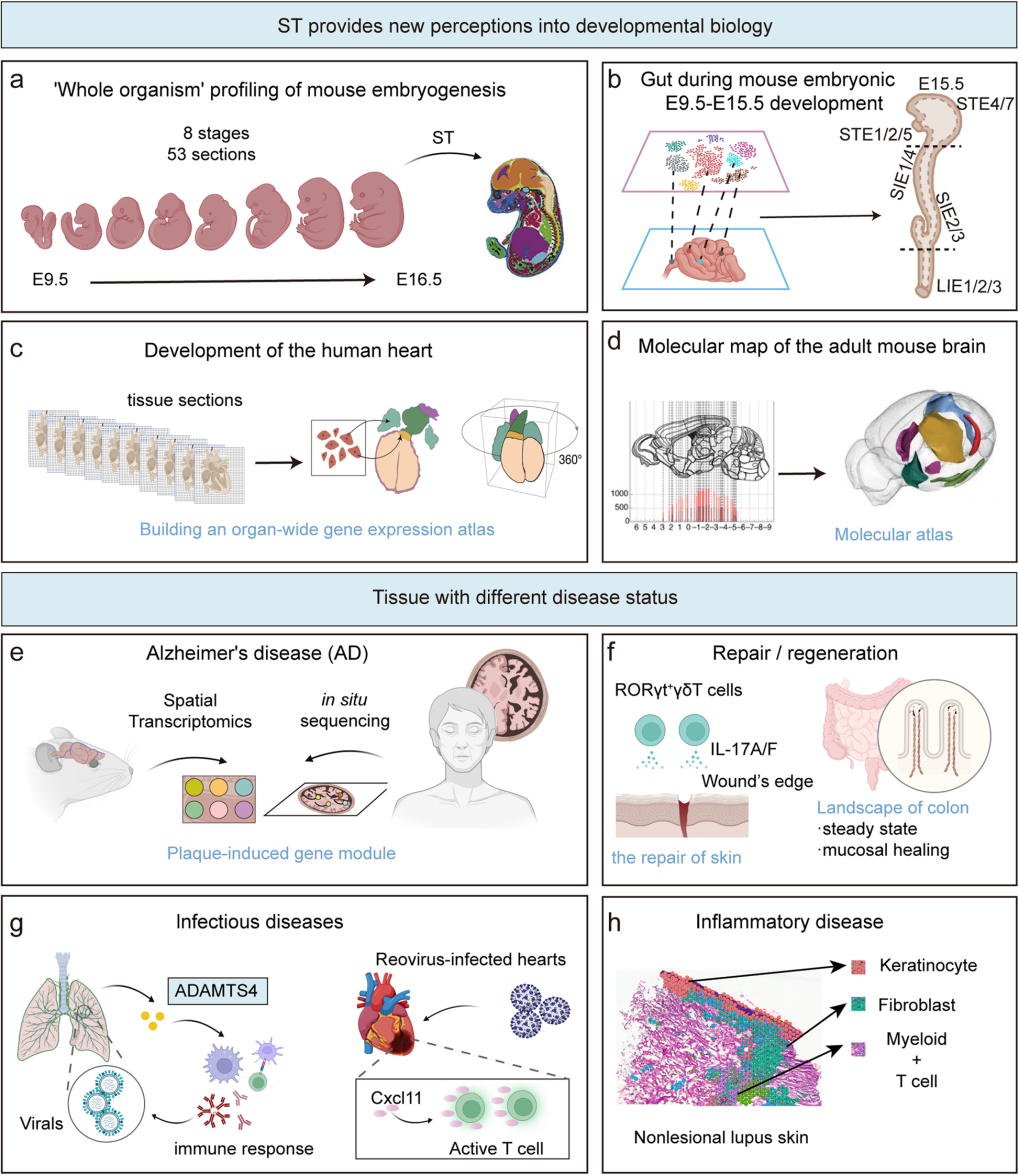
Spatial transcriptomics provides new insights into the molecular mechanisms underlying organ and embryonic development and human pathological tissues. **a** Stereo-seq maps spatiotemporal transcriptome dynamics in developing mouse embryos, detailing tissue-specific identities at different stages. **b** Using scRNA-seq and ST, the spatiotemporal landscape of the mouse stomach and intestine was established, unveiling distinct cell clusters and their interactions responsible for gastric compartmentalization. **c** ST provides a detailed transcriptional map of cell types within the developing heart at three stages, pinpointing cell-type-specific gene expression within distinct anatomical regions. **d** By utilizing coronal brain sections covering the entire anterior–posterior axis, ST generates a comprehensive molecular map of the mouse brain. **e** Combining various ST techniques using both mouse and human tissue samples unveiled widespread changes in the transcriptome and co-expression networks caused by amyloid plaques in (AD). **f** Employing ST into tissue repair uncovers molecular compartmentalization and transcriptome alterations in both steady state and mucosal healing. **g** ST elucidate the dynamic gene expression in the tissue during pathogen infection. **h** The complex cellular structure and heterogeneity of cutaneous lupus erythematosus has been characterized by integrating scRNA-seq and ST analysis in autoimmune diseases

Organogenesis is a complex process that involves dramatic changes in gene expression and frequent shifts in cell fate within a short time span, resulting in the formation of various organs and cell types. Errors in this critical stage can cause serious birth defects, so it is important to examine the spatiotemporal expression patterns during organogenesis to better understand the mechanisms of disease (Fig. 4).

To explore the spatial coordination of embryo development, researchers have developed Stereo-seq [118], a method that preserves single cell resolution while capturing spatial heterogeneity at larger scales. Stereo-seq was used to generate the first spatiotemporal “whole organism” profile of mouse embryogenesis, revealing the tissue- and location-specific transcriptional regulation and cell fate determination over time. Similarly, Asp et al. obtained the first spatiotemporal resource of human developing heart, which comprehensively characterized the dynamic gene expression across time and space at an organ-wide level during human cardiac morphogenesis [119]. Zhao et al. constructed a comprehensive spatiotemporal transcriptome map of the tissues developing along the gut axis during mouse embryonic development from E9.5 to E15.5, and showed that mesenchymal-epithelial interactions regulate key developmental events and cell fate decisions [120], providing new insights into genetic defects in neonatal disease and gut development. Zeng et al. elucidated the evolving molecular and cellular terrain of early gastrulation and nervous system development. Through an analysis of spatial transcriptomic profiles of human embryos, they unveiled processes such as cell diversification, spatial arrangement of neural tube cells, and crucial signaling pathways implicated in cellular transformation [121]. Arutyunyan et al. offer an in-depth investigation into postimplantation trophoblast differentiation using spatial multiomics analysis, providing insights that can guide the development of experimental models for studying early pregnancy in the human placenta [122].

Likewise, spatiotemporal profile of developmental tissue from human [119, 123–126], mouse [118, 120, 127, 128], zebra fish [129], fruit fly [130], and chicken [131] have been generated, which offer valuable resources for developmental biology and facilitate the understanding of abnormal mammalian organogenesis.

### Homeostatic tissue architecture

Once developed, tissues/organs under homeostasis maintain an ordered spatial architecture. ST is widely used to analyze the molecular spatial structure of tissues and to create biomolecular maps for clinical and biological research (Fig. 4). ST-based approaches can map the entire brain [132–134] or specific regions such as olfactory bulb [40, 135], dorsolateral prefrontal cortex [136, 137], hippocampus [138] and arcuate nucleus [139] without tissue dissociation of delicate neurons. Ortiz et al. created a molecular map of the adult mouse brain by hybridizing 75 coronal sections from one hemisphere onto the ST array [140]. Mapping human dorsolateral prefrontal cortex uncovered spatial patterns in genes associated with schizophrenia and autism [141], suggesting possible mechanisms of genetic susceptibility to these disorders. Recently, Chen et al. created an extensive 3D single-cell atlas of the cynomolgus monkey cortex. This atlas offers insights into the cellular and molecular underpinnings of primate brain evolution, development, and pathogenesis [142].

To explore the spatial coordination of human immune development, Suo et al. presented a spatial atlas of human immune system across prenatal hematopoietic organs and characterized the developing immune system [143]. They also functionally validated the properties of human prenatal innate-like B and T cells. Likewise, Gao et al. constructed a spatial profile of mouse fetal liver to dissect hematopoietic stem cell and multipotent progenitor development and expansion in fetal liver [128]. They discovered novel “pocket-like” units of hematopoietic stem and multipotent progenitor cells, which may affect the efficacy of stem cell therapies. ST-based approaches have also been applied to assess the homeostasis of healthy tissues such as prostate [144], lung [145–148], liver [149–152], kidney [153–155], intestine [156, 157], heart [158–161], endometrium [162], embryo [163, 164], muscle [165], adipose [166] and bone [167]. These valuable data resources will enhance our understanding of how cell populations collaboratively shape tissue morphology. With expected future advances in the spatial genomics field, the increased resolution and capture size will enable detailed investigation of rare cell populations across spatial domains.

### Disease/pathological conditions

ST is also a powerful tool for elucidating the molecular mechanisms of pathogenesis by dissecting localized gene expression in normal and abnormal regions of tissue (Fig. 4). Alzheimer’s disease (AD) is a devastating neurological disorder characterized by progressive loss of mental skills, cognition, and physical function. To understand the gene alterations associated with amyloid plaques in AD, several studies used ST to reveal genome-wide transcriptomic changes and co-expression networks induced by amyloid plaques [168, 169]. Similarly, Navarro et al. generated spatial profiles of AD mouse models in early phase and improved our understanding of gene expression perturbation in hippocampus and olfactory bulb during disease progression [170]. ST has also been applied to other neurological diseases, such as brain [171–173] or spinal cord [174] injury as well as neurodegenerative diseases [175–177]. For example, Maniatis et al. conducted a spatiotemporal profile of mouse spinal cord over the course of amyotrophic lateral sclerosis [175], revealing the molecular mechanisms regulating sub-populations of microglia and astrocytes involved in each stage of disease progression. The spatial profile of dysfunctional brains facilitates the discovery of novel mechanisms of brain diseases, leading to the development of new molecular biomarkers/targets.

In addition to the central nervous system, spatially resolved analysis reveals spatial heterogeneity and region-specific cellular crosstalk in tissue regeneration. To uncover the molecular regionalization of the colon repair process, Sara et al. exploited ST to characterize the transcriptomic landscape of colon at steady state and mucosal healing [178]. They revealed that drastic transcriptomic changes occur in the distal colon such as the JAK-STAT and TNF-α pathway, but not in the proximal colon. Ben-Moshe et al. analyzed the spatial and temporal dynamics of the coordinated response of multiple cell types during liver regeneration [179]. Frede et al. discovered the expansion of an IFN-induced B cell subset during mucosal healing, with the depletion of this B cell population leading to improved mucosal healing following intestinal injury [180]. Likewise, ST tracks the interactions between lymphocytes and wound edge epithelium of the skin, identifying RORγt^+^ γδ T cell-derived IL-17A as an essential mediator for skin repair [181].

Similarly, ST analysis reveals the regional heterogeneous muscle pathology during injury and generated the pilot datasets to investigate the molecular dynamics of muscle regeneration [165, 182–184]. Remarkably, McKellar et al. dveloped a spatial total RNA-sequencing approach that captures coding, noncoding and viral RNAs, and identified spatially expressed noncoding RNAs in skeletal muscle regeneration [182].

ST also maps the pathogenesis of infectious diseases. To characterize and understand the host-microbe interaction across space, Gracia Villacampa et al. performed spatial genome-wide RNA analysis on the lung of the SARS-CoV-2 patient and identified distinct spatial expression modules and the coordinated enrichment of specific cells associated with infection by NNMF method [173]. Furthermore, Mothes et al. revealed the significance of activated adventitial niches in driving prolonged lung immunopathology by ST, linked to chemokine up-regulation, endothelial-to-mesenchymal transition, tissue fibrosis, CCR7-expressing exhausted T cell accumulation, and the formation of lymphoid aggregates and ectopic lymphoid structures [185]. In another study, Boyd et al. used scRNA-seq and ST to elucidate the dynamic gene expression of lung under infection and revealed that lung fibroblasts are critical for coordinating immune response at the site of infection by producing extracellular matrix remodeling enzymes ADAMTS4 [186].

Most studies on spatial host-microbe interactions focus on the gene expression of host due to the inability to capture the non-polyadenylated transcripts. To overcome this limitation, McKellar et al. demonstrated a spatial total RNA-seq by adding poly(A) tails to RNAs in situ and profiled the coordinated heterogeneity of heart under viral-induced myocarditis [182]. Moreover, Saarenpää et al. [187] and Lötstedt et al. [188] developed spatial host-microbiome sequencing by simultaneously capturing mRNA and 16S sequences. These improvement versions of ST reveal the spatial organization of microbes within the hosts as well as the coordinated immune response of host upon infection.

The ability of ST to investigate the signatures of disease-driving cells versus cells from the normal anatomical regions offers insights into the pathogenic microenvironment in inflammatory disease. For example, Ferreira and colleagues utilized ST to identify localized renal cell types and immune cell to uncover potential chemotactic signals underlying the pathogenesis [189, 190]. Billi et al. performed integrated scRNA-seq and ST analysis on skin of cutaneous lupus erythematosus (CLE) patients, and demonstrated that the accumulation of CD16^+^ DCs drives CLE pathogenesis [191]. Krausgruber et al. conducted spatial transcriptomics on patient-derived granulomas, uncovering a network of pathogenic macrophages, T cells, and fibroblasts within these structures [192]. Li et al. conducted a spatially resolved multiomics analysis on primary biliary cholangitis (PBC) samples, revealing the presence of a distinctive population of DUOX2^+^ACE2^+^ small cholangiocytes that play a role in PBC pathogenesis [193]. To understand oral chronic disease pathogenesis, Caetano et al. performed spatial transcriptomic analysis on human and mouse oral mucosa, and defined highly specialised epithelial and stromal compartments describing location-specific immune programs [194]. ST analysis was also conducted in other inflammatory disorders such as arthritis [195], periodontitis [196], IgG4-related disease [197] and psoriasis [198, 199].

ST is also improving our understand of other disease with anatomically distinct regions such as fatty liver disease [200], kidney with diabetic disease [164], heart failure and myocardial infarction [201, 202]. Taken together, ST expanded our understanding of spatially localized disease mechanisms, and identified distinct cell populations driving or being activated by disease.

### Cancer

**Fig. 5:**
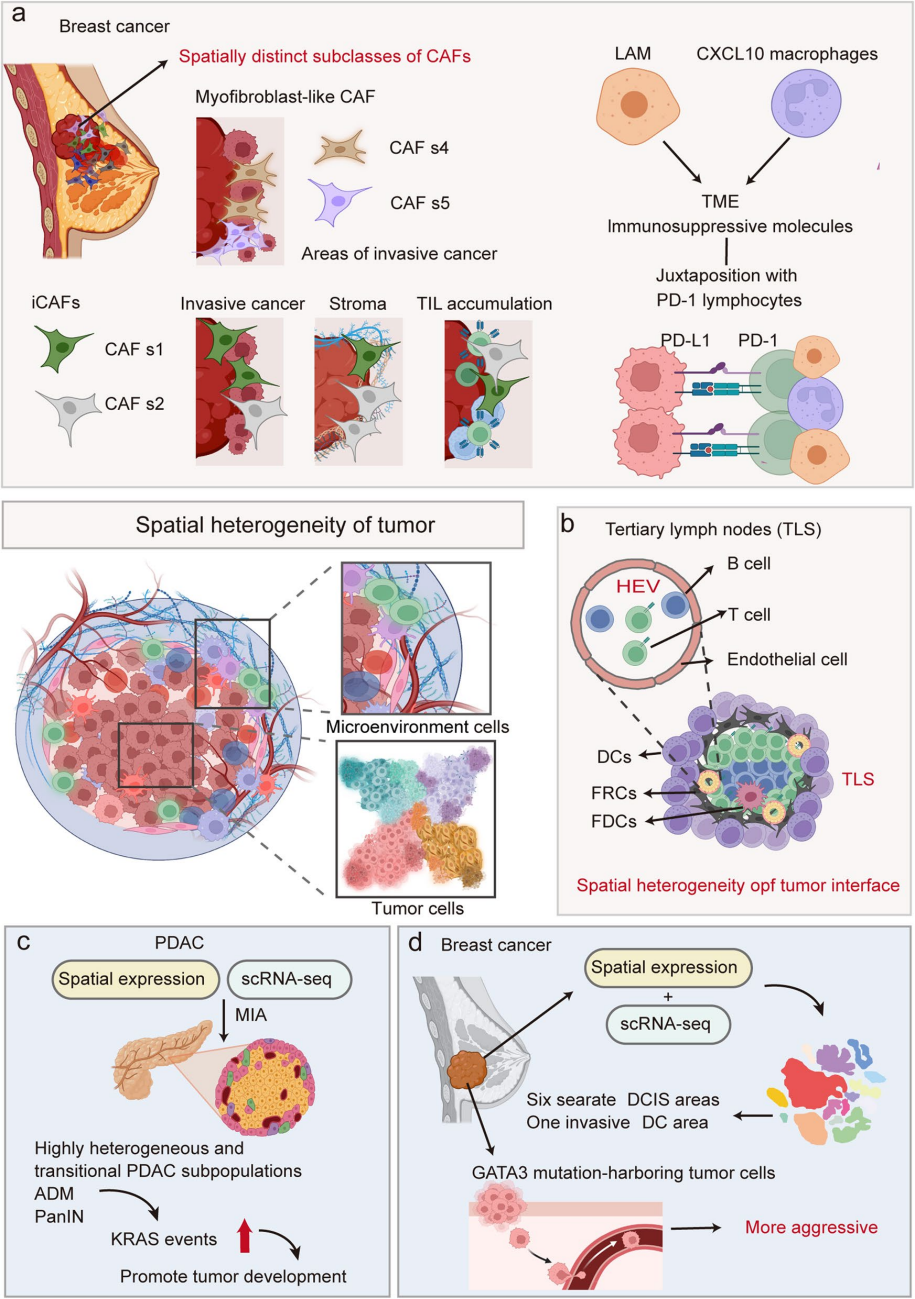
Spatial transcriptomics techniques facilitate the study of tumor microenvironment heterogeneity and tumor heterogeneity. **a** ST examines the diversity within cancer-associated fibroblasts and immunosuppressive molecules within the microenvironment of breast cancer. **b** ST enables the identification and comprehensive exploration of unique tumor microenvironment regions, such as the tumor interface and tertiary lymph nodes. **c** ST analyzes the spatial distribution of PDAC-associated heterogeneity, identifying highly heterogeneous and transitional PDAC subpopulations. **d** ST applied to breast cancer biopsy identified that tumor cells harboring *GATA3* mutations became more invasive, revealing the spatial heterogeneity of breast cancer

ST probably has the widest application in cancer research (Fig. 5). Tumor is a complex ecosystem composed of heterogeneous molecules, cells, and tissues. Intratumor heterogeneity across time and space is a major challenge to cancer therapy, which can be investigated by ST.

The first layer of intratumor heterogeneity is the gene expression and phenotypical variations of tumor cells themselves. Nagasawa et al. dissected the spatial heterogeneity of ductal carcinoma of the breast cells by ST, and identified that *GATA3* mutation-harboring tumor cells underly the progression to invasive cancer [203]. Similarly, ST on breast cancer biopsies identified ductal carcinoma in situ (DCIS) areas with extensive spatial heterogeneity and distinct subclones that cannot be detected by conventional transcriptome analysis [40, 204]. In pancreatic ductal adenocarcinoma (PDAC), deconvolution of spatial transcriptome data identified highly heterogeneous and transitional PDAC subpopulations exhibiting signatures of proliferation, KRAS signaling, cell stress and epithelial-to-mesenchymal transition [205]. Hao et al. analyzed the spatial distribution of PDAC hypoxia-related heterogeneity based on spatial transcriptomics (ST), revealing the localization of highly aggressive subgroups and their changes in hypoxia-related genes [206]. ST on FFPE human prostate cancer revealed that luminal cells in tumors are greatly expanded in the invasive carcinoma region and did not co-localize with basal cells [207, 208]. ST also provides new insights into drug resistance tumor cells. ST analysis of paired primary and recurrent prostate cancer samples demonstrated that treatment-resistant subpopulations are interspersed in apparently benign tissues with unique molecular features [209], driving prostate cancer relapse.

The second layer of intratumor heterogeneity involves the complex interplay between tumor cells and tumor microenvironmental (TME) cells. One area that draws a lot of interest is the tumor-immune cell interactions, which have implications in cancer immunotherapy. In breast cancer, the spatial profiling of the stromal immune niche in tumors provides insights into the regulation of antitumor immunity. Spatially distinct subclasses of cancer-associated fibroblasts colocalize with and may be involved in direct regulation of immune cells [210]. Lipid-associated macrophages and CXCL10^hi^ macrophages as a major source of immunosuppressive cells in the TME, and spatial analysis revealed their juxtaposition with PD-1^+^ lymphocytes [210]. Within lung adenocarcinoma, spatial transcriptomics unveils an upregulation of genes associated with VEGF and CCR2 signaling in response to Treg cell depletion. Notably, short-term VEGF blockade significantly enhances control over the progression of PD-1 blockade-resistant lung adenocarcinoma [211]. Likewise, Ozato et al. demonstrated that tumor cells trigger the expression of human leukocyte antigen G (HLA-G), leading to the generation of secreted phosphoprotein 1 (SPP1)^+^ macrophages, which in turn bestow colorectal cancer cells with anti-tumor immune properties [212].

Aside from tumor-immune cell interactions, ST also reveal the interplay between tumor and stromal cells. In esophageal squamous-cell carcinoma (ESCC), Chen et al. employed spatial transcriptomics to characterize the progression of ESCC tumorigenesis across different stages, highlighting the gradual depletion of ANXA1 in epithelial cells along the tumorigenesis process, which in turn promotes ESCC by triggering the formation of cancer-associated fibroblasts [213]. In cutaneous squamous cell carcinoma, Khavari group orthogonally integrated single-cell and high-dimensional spatial data of normal and diseased tissues, revealing that tumor-specific keratinocytes as hubs of intercellular communication and observed multiple hallmarks of potential immunosuppression [214]. In colorectal cancer, tumor-specific FAP-positive fibroblasts and SPP1-positive macrophages are closely localized, which may contribute to poor patient survival [215]. ST on PDAC also revealed colocalization of stress-responsive cancer cells and inflammatory fibroblasts [216]. Similar cancer-TME interactions have been revealed by ST in ESCC [217], neuroblastoma [218], and malignant gliomas [219–221].

The third layer of intratumor heterogeneity involves special spatial regions such as the tumor-normal interface and tertiary lymph like structures (TLS), forming unique tumor niches implicated in cancer progression. In gastric cancer, Sun et al. performed spatially resolved multi-omics to identify an immune cell-dominated 'tumor-normal interface' region characterized by unique transcriptional signatures and notable immunometabolic changes [222]. In liver cancer, Wu et al. investigated the tumor ecosystems and cell interactions within an "invasive zone" around the liver tumor border, and identified the damaged hepatocytes with high expression of serum amyloid A1 and A2 which was associated with a worse prognosis [223]. In kidney cancer, scRNA-seq and ST analysis of cells at the tumor-normal interface versus the tumor core revealed an epithelial-mesenchymal transition meta-program highly enriched at the tumor-normal interface that co-localizes with IL1B-expressing macrophages [224]. Anderson et al. performed spatial profiling of HER2-positive breast tumors, mapped tumor-associated cell types to find TLS, and constructed a predictive model to infer presence of tertiary lymphoid-like structures [225]. Similarly, Wu et al. identified a TLS-50 signature to accurately locate TLS in primary liver cancer [226].

### Conclusion and perspective

**Fig. 6:**
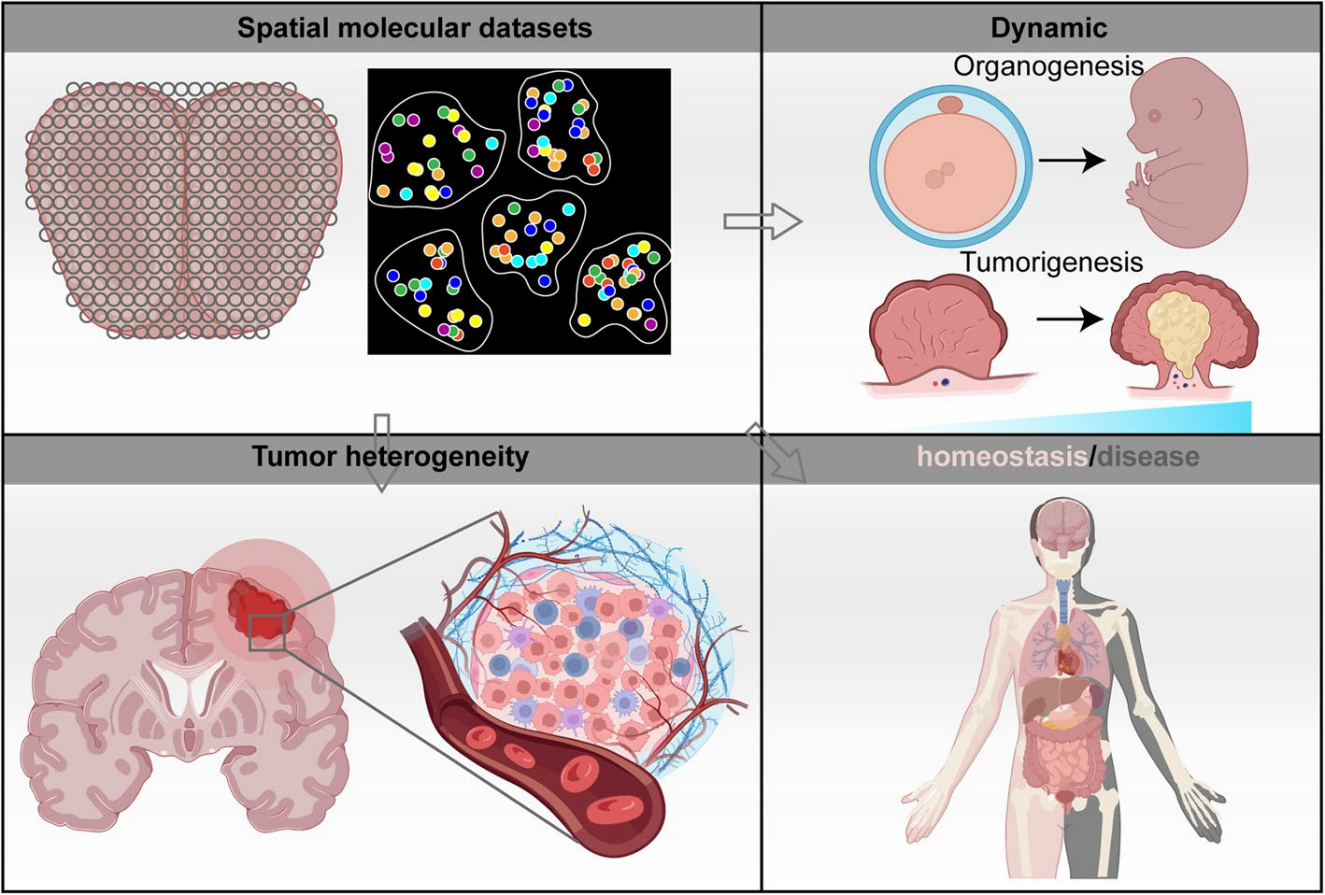
The future directions of spatial genomics. The ongoing advancement of spatial genomics techniques is driving research in areas such as tissue homeostasis, diseases, tumor and embryo development, and tumor heterogeneity. These advancements hold the potential to offer valuable insights into both biological understanding and clinical applications

In summary, ST is a powerful method for mapping gene expression in tissues/organs that can provide new insights into the mechanisms of development, homeostasis and disease (Fig. 6).

Despite the explosion of technological innovation of ST in recent years, some of the technical limitations of current ST approaches remain. The first challenge is how to improve resolution without compromising throughput. The spatial resolution of current methods can vary from subcellular to regional level, and there’s often a trade-off between resolution and throughput. Microdissection or in situ image-based sequencing can achieve high spatial resolution but at the expense of low throughput and high complexity. Conversely, techniques like spatial barcoding-based approachs offer improved scalability and throughput, albeit at the expense of diminished spatial resolution. The development of high-throughput, enhanced resolution spatial barcoding techniques such as Stereo-seq is a step in the right direction, but there is still room for improvement in terms of the spot size, spot distance, and transcripts detected per spot to achieve single-cell, subcellular spatial profile.

The second challenge is the diverse range of methodologies that leads to a multitude of file formats and data structures. This complexity makes data and protocol sharing more difficult. Therefore, there is a growing need to develop universally applicable file formats and establish standardized pipelines for data storage, access, and cross-dataset integration.

The third challenge involves harmonizing spatially resolved data across both intra-omics and cross-omics layers. When considering the integration of spatially resolved omics data within the same layer, a range of complexities emerges due to technical concerns during sample collection. These challenges encompass issues like possible tissue gaps, distortions introduced during sectioning, tears, and the presence of variations in both biological and technical facets, such as differing resolutions across various ST methodologies. In the context of cross-omics layers, the integration of multi-omics data poses a significant challenge. This is particularly pronounced due to the considerable variation in feature counts between different modalities (for instance, proteins versus transcripts) and the existence of distinct statistical distributions.

These technical limitations pose challenges for spatial transcriptomics data generation, processing, analysis and interpretation. Therefore, researchers need to carefully consider the trade-offs between different methods and choose the most suitable one for their specific biological questions and experimental settings.

Accordingly, the future directions of ST are to improve the spatial resolution, gene coverage, sensitivity, and reducing complexity of existing methods. We could develop new probes, arrays, sequencing strategies and imaging systems to increase the accuracy and throughput of ST. We also need to develop new computational tools to improve data processing, integration, visualization and interpretation, as well as inferring cell–cell interactions, spatial patterns and regulatory networks. Integrating ST with other omics methods, such as proteomics, metabolomics or epigenomics, could obtain a more comprehensive view of biological systems at multiple levels of resolution. Ultimately, we could develop spatiotemporal single-cell omics methods that can capture the dynamics and positioning of molecular profiles in living tissues.

## Data Availability

Not applicable.
